# Timing of SGLT2i initiation after acute myocardial infarction

**DOI:** 10.1186/s12933-023-02000-5

**Published:** 2023-09-30

**Authors:** Dirk von Lewinski, Ewald Kolesnik, Faisal Aziz, Martin Benedikt, Norbert J. Tripolt, Markus Wallner, Peter N. Pferschy, Friederike von Lewinski, Nora Schwegel, Rury R. Holman, Abderrahim Oulhaj, Deddo Moertl, Jolanta Siller-Matula, Harald Sourij

**Affiliations:** 1 Department of Internal Medicine, Division of Cardiology, Medical University of Graz, Auenbruggerplatz 15, 8036 Graz, Austria; 2 Department of Internal Medicine, Division of Endocrinology and Diabetology, Medical University of Graz, Auenbruggerplatz 15, 8036 Graz, Austria; 3 Interdisciplinary Metabolic Medicine Trials Unit, Medical University of Graz, Graz, Austria; 4 Radcliffe Department of Medicine, University of Oxford, Oxford, UK; 5 Department of Epidemiology and Population Health, College of Medicine and Health Sciences, Khalifa University, Abu Dhabi, UAE; 6 Karl Landsteiner University of Health Sciences, 3050 Krems, Austria; 7 Department of Internal Medicine 3, University Hospital St. Poelten, 3100 St. Poelten, Austria; 8 Department of Cardiology, Medical University of Vienna, Vienna, Austria; 9 Department of Internal Medicine, Division of Endocrinology and Diabetology, Medical University of Graz, Graz, Austria

**Keywords:** Myocardial infarction, SGLT2i, Timing, Clinical trial

## Abstract

**Background:**

Pharmacological post-MI treatment is routinely initiated at intensive/cardiac care units. However, solid evidence for an early start of these therapies is only available for dual platelet therapy and statins, whereas data on beta blockers and RAAS inhibitors are heterogenous and mainly limited to STEMI and heart failure patients. Recently, the EMMY trial provided the first evidence on the beneficial effects of SGLT2 inhibitors (SGLT2i) when initiated early after PCI. In patients with type 2 diabetes mellitus, SGLT2i are considered “sick days drugs” and it, therefore, remains unclear if very early SGLT2i initiation following MI is as safe and effective as delayed initiation.

**Methods and results:**

The EMMY trial evaluated the effect of empagliflozin on NT-proBNP and functional and structural measurements. Within the Empagliflozin group, 22 (9.5%) received early treatment (< 24 h after PCI), 98 (42.2%) within a 24 to < 48 h window (intermediate), and 111 (48.1%) between 48 and 72 h (late). NT-proBNP levels declined by 63.5% (95%CI: − 69.1; − 48.1) in the early group compared to 61.0% (− 76.0; − 41.4) in the intermediate and 61.9% (− 70.8; − 45.7) in the late group (n.s.) within the Empagliflozin group with no significant treatment groups—initiation time interaction (p_int_ = 0.96). Secondary endpoints of left ventricular function (LV-EF, e/e`) as well as structure (LVESD and LVEDD) were also comparable between the groups. No significant difference in severe adverse event rate between the initiation time groups was detected.

**Conclusion:**

Very early administration of SGLT2i after acute myocardial infarction does not show disadvantageous signals with respect to safety and appears to be as effective in reducing NT-proBNP as well as improving structural and functional LV markers as initiation after 2–3 days.

## Introduction

Timely reperfusion by primary percutaneous coronary intervention (PCI) is a cornerstone in the treatment strategy for myocardial infarction (MI) [1] and its widespread implementation has significantly reduced mortality [2]. Pharmacological post-MI treatment is routinely initiated within the first 48 h including dual platelet therapy, statins, beta blockers, and renin–angiotensin–aldosterone system (RAAS) inhibitors. However, solid evidence for an early start of these therapies and guideline recommendations are only available for dual platelet therapy and statins [1]. Whereas, data on beta blockers is heterogenous and mainly limited to STEMI patients [1, 3]. Data on RAAS inhibition are even more scarce and general recommendations are limited to patients with concomitant heart failure [1, 4]. Recently, the EMpaglifozin in acute MYocardial infarction (EMMY) trial provided the first evidence on the beneficial effects of SGLT2 inhibitors (SGLT2i) when initiated within 72 h after PCI in addition to guideline-directed therapy [5]. In patients with type 2 diabetes mellitus, SGLT2i are considered “sick days drugs” with recent diabetes guidelines suggesting avoidance of SGLT2i in severe illness and even recommend against routine use of these drugs during hospital stays until safety and effectiveness are established [6]. Data for SGLT2i treatment in ICU patients are generally very limited but was shown to be feasible and without increasing adverse events in a recent pilot trial in diabetic patients [7]. Current MI guidelines recommend close monitoring of kidney function in patients taking SGLT2i for at least three days post-PCI [1]. It therefore remains unclear if very early SGLT2i initiation (< 24 h after PCI) following an acute MI is as safe and effective as its delayed initiation. This is of practical relevance since most patients spend their first 24 h in an ICU/CCU and chronic treatments are routinely established there.

## Methods

A secondary analysis of the EMMY trial was conducted. The methodological details and primary results of the trial have been published recently [5]. In short, the EMMY was a multicenter, randomized (1:1 ratio), double-blind, and placebo-controlled trial investigating the effect of Empaglifozin (10 mg once daily), administered for 26 weeks in patients with AMI (n = 476 patients). NT-proBNP changes were the primary outcome with functional and structural measurements using echocardiography being secondary outcomes. The trial enrolled patients within 72 h after AMI (creatinine kinase > 800U/l) and who already underwent percutaneous coronary intervention. Patients had to be aged 18–80 years, haemodynamically stable, and had a blood pressure > 110/70 mmHg. Patients with other types of diabetes, a blood pH < 7.32, haemodynamic instability, acute urinary tract or genital infections, on current SGLT2i therapy, or those who received the SGLT2i treatment within four weeks before enrolment were excluded from the trial.

The study was approved by the relevant regulatory authorities, by the Ethics Committee of Medical University of Graz, Austria (EK 29-179 ex 16/17; EudraCT 2016-004591-22) and was registered on ClinicalTrials.gov (NCT03087773). The trial was conducted in full conformity with the 1964 Declaration of Helsinki and all subsequent revisions, as well as in accordance with the guidelines laid down by the International Conference on Harmonization for Good Clinical Practice (ICH GCP E6 guidelines).

Our analysis compared the effects of very early SGLT2i initiation, (< 24 h after PCI; early) with later timepoints, with respect to primary and secondary outcomes and the EMMY trial safety measures. Linear regression was applied to compare the log-transformed percentage change of each biomarker from 12 to 26 weeks with treatment group, visit, and treatment-by-visit interaction. The analysis was adjusted for baseline values of each marker, age, sex, and diabetes status.

## Results

Within the Empagliflozin group (N = 231 with all biosamples available), 22 (9.5%) received early treatment within < 24 h, 98 (42.2%) within 24 to < 48 h (intermediate), and 111 (48.1%) between 48 and 72 h (late) following the PCI. The treatment initiation timings were not significantly different between Empagliflozin and Placebo groups (p = 0.79). Baseline characteristics of EMMY participants including NT-proBNP, did not show significant differences between those receiving the Empagliflozin treatment early, intermediate, or late (Table 1).

**Table 1:** Characteristics of participants by treatment initiation status

Variable	N	Overall (N = 463)	Treatment initiation			
			< 24 h (N = 41)	24–< 48 h (N = 193)	48–< 72 h (N = 229)	P-value
Treatment, n (%)	463					0.787
Empagliflozin		231 (50)	22 (54)	98 (51)	111 (48)	
Placebo		232 (50)	19 (46)	95 (49)	118 (52)	
Sex, n (%)	463					0.891
Female		81 (17)	7 (17)	32 (17)	42 (18)	
Male		382 (83)	34 (83)	161 (83)	187 (82)	
Age (years), median (IQR)	463	57 (52, 64)	57 (54, 63)	58 (52, 65)	57 (51, 64)	0.363
Body mass index (kg/m^2^), median (IQR)	463	28 (25, 30)	28 (26, 31)	28 (25, 30)	27 (25, 30)	0.280
Type 2 diabetes, n (%)	463	62 (13)	8 (20)	26 (13)	28 (12)	0.451
Systolic blood pressure, median (IQR)	463	125 (117, 131)	126 (121, 135)	125 (115, 130)	125 (118, 131)	0.098
Diastolic blood pressure, median (IQR)	463	78 (74, 85)	78 (75, 82)	77 (74, 82)	78 (74, 86)	0.344
Smoking (active or former), n (%)	463	334 (72)	31 (76)	146 (76)	157 (69)	0.385
Dyslipidemia, n (%)	463	129 (28)	4 (10)	46 (24)	79 (34)	0.001
Hypertension, n (%)	463	193 (42)	13 (32)	74 (38)	106 (46)	0.102
Coronary artery disease, n (%)	463	52 (11)	4 (10)	13 (7)	35 (15)	0.018
History of stroke, n (%)	463	6 (1)	0 (0)	4 (2)	2 (1)	0.555
History of CABG, n (%)	463	2 (0)	1 (2)	0 (0)	1 (0)	0.169
Coronary angiography vessel status, n (%)	463					0.588
1-vessel disease		219 (47%)	15 (37%)	92 (48%)	112 (49%)	
2-vessel disease		161 (35%)	16 (39%)	65 (34%)	80 (35%)	
3-vessel disease		83 (18%)	10 (24%)	36 (19%)	37 (16%)	
History of carcinoma, n (%)	463	23 (5)	4 (10)	7 (4)	12 (5)	0.206
Depression, n (%)	463	24 (5%)	2 (5%)	7 (4%)	15 (7%)	0.436
Laboratory parameters						
NT-proBNP (pg/ml), median (IQR)	444	1345 (754, 2222)	1301 (812, 1971)	1433 (824, 2416)	1198 (696, 2123)	0.097
eGFR (ml/min/1.73 m^2^), median (IQR)	462	92 (78, 101)	94 (79, 101)	93 (80, 103)	90 (78, 100)	0.497
Creatine Kinase (U-L), median (IQR)	462	1695 (1203, 2457)	1623 (1169, 2697)	1687 (1220, 2333)	1775 (1203, 2597)	0.607
Troponin T (ng/l), median (IQR)	444	3056 (2055, 4899)	4945 (3258, 5570)	3029 (2199, 4938)	2808 (1885, 4250)	< 0.001
HbA1c (%), median (IQR)	442	6 (5, 6)	6 (5, 6)	6 (5, 6)	6 (5, 6)	0.112
Total cholesterol (mg/dl), median (IQR)	453	188 (162, 223)	195 (174, 236)	189 (163, 222)	186 (161, 221)	0.180
Triglycerides (mg/dl), median (IQR)	449	124 (92, 174)	115 (62, 179)	123 (90, 167)	126 (98, 178)	0.306
HDL-C (mg/dl), median (IQR)	445	43 (36, 52)	44 (35, 55)	43 (35, 52)	44 (36, 52)	0.673
LDL-C (mg/dl), median (IQR)	449	119 (92, 148)	131 (107, 159)	119 (95, 144)	117 (89, 150)	0.145
Alanine aminotransferase (IU/L), median (IQR)	456	50 (37, 74)	54 (38, 74)	47 (37, 69)	53 (37, 77)	0.434
Aspartate aminotransferase (IU/l), median (IQR)	456	206 (125, 325)	223 (132, 312)	225 (150, 308)	191 (99, 331)	0.097
Gamma glutamyltransferase (IU/l), median (IQR)	453	31 (21, 49)	36 (21, 54)	30 (21, 47)	31 (21, 49)	0.634
Treatment						
ACE-1/ARB, n (%)	463	446 (96)	40 (98)	184 (95)	222 (97)	0.387
ARNI, n (%)	463	9 (2)	0 (0)	3 (2)	6 (3)	0.206
Beta-blocker, n (%)	463	448 (97)	40 (98)	186 (96)	222 (97)	0.257
Mineralocorticoid receptor agonist, n (%)	461	180 (39)	21 (53)	78 (41)	81 (36)	0.103
Loop diuretic, n (%)	463	49 (11)	6 (15)	21 (11)	22 (10)	0.199
Statin, n (%)	463	450 (97)	40 (98)	191 (99)	219 (96)	0.011
Ezetimibe, n (%)	463	59 (13)	6 (15)	22 (11)	31 (14)	0.267
Calcium channel blocker, n (%)	463	20 (4)	3 (7)	11 (6)	6 (3)	0.030
Antiplatelet inhibitory drug, n (%)	463	463 (100)	41 (100)	193 (100)	229 (100)	1.000
Anticoagulant drug, n (%)	463	37 (8)	5 (12)	15 (8)	17 (7)	0.142
Metformin, n (%)	463	41 (9)	5 (12)	19 (10)	17 (7)	0.112
DPP4 inhibitor, n (%)	463	12 (3)	1 (2)	7 (4)	4 (2)	0.100
Sulfonylurea, n (%)	463	3 (1)	0 (0)	1 (1)	2 (1)	0.178
GLP1-RA, n (%)	463	4 (1)	1 (2)	1 (1)	2 (1)	0.072
Insulin, n (%)	463	11 (2)	0 (0)	6 (3)	5 (2)	0.144

Continuous variables are reported as median (IQR) and categorical variables as frequencies (%)

P-values are reported for Kruskal–Wallis test, Chi-square test, or Fischer Exact test

**Fig. 1:**
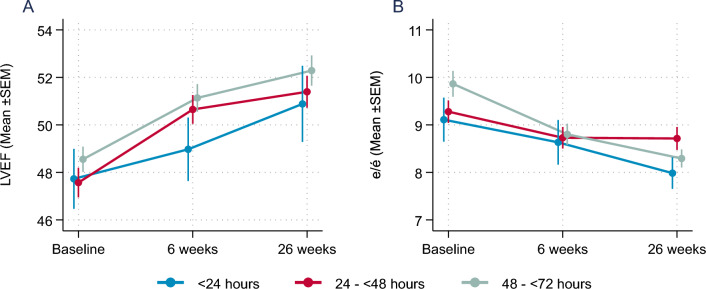
(**A**) Mean±SEM change in LV-EF (left ventricular ejection fraction; %) and (**B**) e/e`(measure of diastolic function) over time

**Fig. 2:**
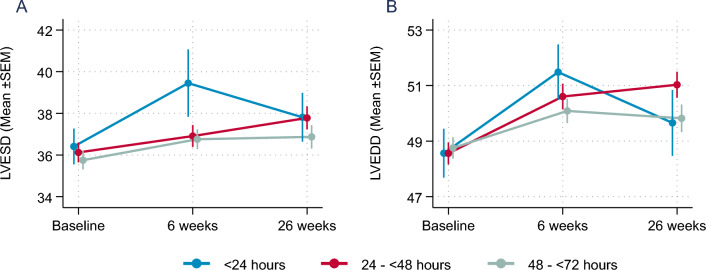
(**A**) Mean±SEM change in left ventricular end systolic volume (LVESD, mm) and (**B**) left ventricular end diastolic volume (LVEDV, mm) over time

NT-proBNP levels declined within the 26 weeks of follow-up by 63.5% (95%CI: − 69.1; − 48.1) in the early group compared to 61.0% (− 76.0; − 41.4) in the intermediate group, and 61.9% (− 70.8; − 45.7) in the late group (n.s.) within the Empagliflozin group with no significant treatment groups—initiation time interaction (p_int_ = 0.96). In secondary endpoints, Left ventricular ejection fraction (LVEF) and increased continuously and was comparable in all three groups (Fig. 1A). Trajectories of e/e` as a measure for the improvement in diastolic function were also comparable between the three groups (Fig. 1B). Similarly, further secondary echocardiography endpoints addressing left ventricular structure (left ventricular end-systolic diameter; LVESD and end-diastolic diameter; LVEDD) with hardly any differences in the change of left ventricular dimensions after 26 weeks (Fig. 2).

No evidence of significant treatment group—initiation time interaction could be detected for any of these parameters.

Compared with the placebo, the treatment effect was numerically most pronounced in the early group. LVEF increased by 7.0% (− 3.4; 13.7) in the Empagliflozin group vs. 1.7% (− 1.7; 12.5) in the placebo group compared to 3.6% (− 2.0; 9.1) vs. 2.2% (− 7.3; 10.6) in the intermediate and 0.0% (− 5.5; 9.1) and 3.4% (− 3.9; 12.1) in the late group, respectively. Similarly, LVESD decline was the most pronounced in the early treatment group − 9.8 ml (− 14.3; 1.1) for Empagliflozin vs. − 2.4 ml (− 24.1; 9.0) for placebo and − 3.3 ml (− 17.8; 5.8) vs. 3.5 ml (− 8.8; 22.2) in the intermediate and − 3.5 ml (− 13.6; 14.0) vs. 0.0 ml (− 11.9; 17.8) in the late group. However, none of these differences reached statistical significance (Table 2).

**Table 2:** Percentage change in cardiac markers from Baseline to visit 4

	Treatment Initiation				
	< 24 h (N = 41)	24–48 h (N = 193)	> 48– < 72 h (N = 229)	P-value	P_interaction_
	Median (IQR)	Median (IQR)	Median (IQR)		
LVEF (%)					
Empagliflozin	7.0 (− 3.4; 13.7)	3.6 (− 2.0; 9.1)	0.00 (− 5.5; 9.1)	0.239	0.550
Placebo	1.7 (− 1.7; 12.5)	2.2 (− 7.3; 10.6)	3.4 (− 3.9; 12.1)	0.898	
E/é					
Empagliflozin	− 9.5 (− 11.3; 9.6)	− 4.9 (− 22.4; 8.9)	− 4.2 (− 18.6; 7.7)	0.664	0.863
Placebo	− 5.6 (− 10.0; − 0.3)	1.1 (− 11.5; 17.3)	− 1.1 (− 14.3; 13.5)	0.720	
LVESD (mm)					
Empagliflozin	− 2.9 (− 9.8; 6.1)	2.6 (− 5.4; 13.5)	0.00 (− 8.3; 9.7)	0.225	0.088
Placebo	1.3 (− 18.9; 7.5)	3.0 (− 5.6; 8.8)	0.00 (− 7.0; 9.5)	0.763	
LVEDD (mm)					
Empagliflozin	− 2.9 (− 10.3; 3.8)	0 (− 6.3; 5.4)	0.00 (− 4.9; 4.0)	0.998	0.622
Placebo	− 1.8 (− 9.5; 1.8)	1.7 (− 3.6; 4.7)	0.00 (− 6.0; 4.9)	0.158	

*LVEF* Left ventricular ejection fraction, *LVESD* left-ventricular end-systolic diameter, *LVEDD* left ventricular end-diastolic diameter

P-value: P-value for linear regression for each treatment groups, adjusted for baseline value of each marker, age, sex, and diabetes status

P_interaction_: P-value for interaction between treatment initiation time and treatment groups

The EMMY trial counted 72 Serious Adverse Events (SAEs). No significant differences in the event rate (early: 17.1%, intermediate: 13.5%, late: 11.8%, p = 0.598) or the median time to SAE (early: 77 (40–144) days: intermediate: 59 (18–154), late: 112 (32–167); p = 0.384) between the initiation groups were detected. Moreover, no treatment discontinuation due to hypotension, renal failure, or ketoacidosis was reported in any of the three groups.

## Discussion

Very early administration of SGLTi Empagliflozin after AMI does not show disadvantageous signals with respect to safety and appears to be as effective in reducing NT-proBNP as well as improving structural and functional LV markers as initiation after 2–3 days.

Mechanisms underlying the beneficial effects of SGLT2i in cardiovascular disease are widely discussed and obviously multifactorial [8]. The findings, however, seem to depend on the models used and only limited data are available from human tissue and trials. Emerging evidence indicates the beneficial effects of SGLT2i therapy in patients with severe coronary artery disease [9] and ACS [10, 11]. A study in diabetic patients with non-obstructive multivessel disease depicted a large reduction of a composite endpoint indicating cardiovascular disease events, hospital admissions for heart failure, and ischemic cardiovascular events accompanied by significantly lower inflammation parameters in the SGLT2i treated group after 1 year. All patients had invasive imaging at baseline and after 12 months. The SGLT2i treated group was characterized by a thicker minimum width of the fibrous cap and a smaller lipid arch representing more stable plaque [9]. With respect to myocardial infarction patients with diabetes, a reduced incidence of in-stent restenosis-related events was shown using the data of a prospective registry. This effect was independent of glycemic control [10]. Confirmative data derived from an international registry of MI patients with diabetes reported significantly lower in-hospital cardiovascular deaths, arrhythmic burden, and acute kidney disease in patients who are hospitalized and further treated with SGLT2i after MI [11].

As shown in the EMMY trial cohort, AMI results in increased inflammation over time but this trajectory is not impacted by empagliflozin treatment [12]. In addition, Trimethylamine N-oxide (TMAO) considered to be involved in pro-atherogenic pathways has been shown to rapidly increase after MI and maintain elevated levels throughout the 26-week observation period with even higher levels in the Empagliflozin group [13].

Although not significantly different from other treatment initiation times, the median baseline NT-proBNP level was the highest in patients randomized at 24 to 48 h after PCI. This observation represents rather the trajectory of NT-proBNP levels after MI than differences in infarct size or severity [14, 15].

The results of two large outcome trials, DAPA-MI and EMPACT-MI [16], for SGLT2i treatment after MI will soon be available and will expand on the evidence of this new therapy concept. However, the SGLT2i treatment initiation happens significantly later in these trials (within 14 days for Empagliflozin and within 10 days after MI for Dapagliflozin). Thus, only limited data will be provided by these trials with respect to the very early phase. Likewise, the EMPULSE trial for Empagliflozin in patients with acute heart failure [17] initiated treatment > 24 h after hospitalization.

This is in line with only limited data on very early initiation post-MI for established therapies such as beta blockers and RAAS inhibitors. A recent meta-analysis indicates a preference for ARNIs compared to ACEI/ARBs but recommends initiation within 24 h after MI for the latter group [18]. Of note, almost all data for very early administration derived from STEMI patients with reduced baseline ejection fraction. In EMMY, predominantly STEMI patients were included (86%), but baseline LV-EF was only slightly reduced (average 48.2 ± 8.2%).

As shown in numerous previous cardiovascular trials, SGLT2i are equally effective in patients with or without diabetes. Safety concerns of this drug class in severely ill or hospitalized patients were addressed by current diabetes guidelines, [6] but also recently challenged based on accumulating data indicating an overall low risk of ketoacidosis, particularly in those people without diabetes, in contrast to robust positive cardiovascular effects [19].

This is supported by the data from the EMPULSE trial [17] with a median treatment initiation on the third day of hospitalization for acute heart failure and thus far providing the best evidence on the early administration of SGLT2i in severely ill cardiovascular patients. Empagliflozin treatment resulted in a higher clinical benefit than placebo with respect to efficacy parameters integrated in a win-ratio and no safety issues were reported. Our analysis extends this finding and suggests safety and efficacy to an immediate SGLT2 inhibitor initiation in people with MI.

## Data Availability

The data underlying this article will be shared on reasonable request to the corresponding author.
